# Disparities and resilience: analyzing online Health information provision, behaviors and needs of LBGTQ + elders during COVID-19

**DOI:** 10.1186/s12889-022-14783-5

**Published:** 2022-12-13

**Authors:** Huizi Yu, Lizhou Fan, Anne J. Gilliland

**Affiliations:** 1 University of Michigan, Ann Arbor, MI USA; 2 University of California, Los Angeles, CA USA

**Keywords:** COVID-19, Elders, Health disparities, Health information, LGBTQ+

## Abstract

**Background:**

Prior studies indicate that older members of LGBTQ+ communities have specific health provision and health information needs related to coping with COVID-19, its long-term effects, and the social and economic impact of the pandemic. This study addresses the issue of a lack of timely, complete, and high-quality data about this population’s healthcare and healthcare information needs and behaviors. Recognizing also that this is a diverse population made up of multiple communities and identities with different concerns and experiences, this research seeks to develop and refine a method that can provide additional nuanced data and insights that can support improved and more closely targeted health interventions and online information provision.

**Methods:**

We use computational discourse analysis, which is based on NLP algorithms, to build and analyze a digital corpus of online search results containing rich, wide-ranging content such as quotes and anecdotes from older members of LGBTQ+ communities as well as practitioners, advice, and recommendations from policymakers and healthcare experts, and research outcomes. In our analysis, we develop and apply an innovative disparities and resilience (D&R) framework to identify external and internal perspectives and understand better disparities and resilience as they pertain to this population.

**Results:**

Results of this initial study support previous research that LGBTQ+ elders experience aggravated health and related social-economic disparities in comparison to the general population of older people. We also find that LGBTQ+ elders leverage individual toughness and community closeness, and quickly adapt mentally and technologically, despite inadequate social infrastructure for sharing health information and elders’ often low social economic status. The methods used therefore are able to surface distinctive resilience in the face of distinctive disparities.

**Conclusions:**

Our study provides evidence that methodological innovation in gathering and analyzing digital data relating to overlooked, disparately affected, and socially and economically marginalized intersectional communities such as LGBTQ+ elders can result in increased external and self-knowledge of these populations. Specifically, it demonstrates the potential of computational discourse analysis to surface hidden and emerging issues and trends relating to a multi-faceted population that has important concerns about public exposure in highly timely and automated ways. It also points to the potential benefits of triangulating data gathered through this approach with data gathered through more traditional mechanisms such as surveys and interviews.

**Trial registration:**

Not Applicable.

## Introduction

Since the World Health Organization (WHO) declared a COVID-19 pandemic on March 11, 2020, the novel coronavirus strand Severe Acute Respiratory Syndrome Coronavirus-2 (SARS-CoV-2) has spread rapidly with unprecedented effects across the entire world. This acute respiratory disease ranges widely in its impact on physical and mental health. It may be asymptomatic or cause mild to severe illness and death [1]. It may also cause long-term physical and mental health conditions even after the initial recovery [2]. Attempting to flatten the curve of COVID-19 cases and deaths, state governments dynamically issued a diversity of social distancing measures and lockdown mandates. However, these precautions contributed to additional emotional and economic stress and other unanticipated mental and psychological issues for many members of the public [3]. These issues have been shown to have had differential impacts on populations depending on their nationality, ethnicity, age, gender, and sexuality [4–9]. One often overlooked and complex population that has particular health provision and health information needs related to coping with the disease, its long-term effects, and the social and economic impact of the pandemic, comprises older people who identify as members of LGBTQ+ communities. Several factors in addition to the variation in identities and experiences of this population make developing knowledge about their needs a challenging problem. These include the newness of the disease, the rapid evolution of the virus, and the urgency of developing effective responses and treatments. This situation calls for data gathering and analysis methods that are as rapid and as customizable as possible. Additionally, the often undisclosed or societally stigmatized status of LGBTQ+ identifying individuals calls for the use of data-gathering methods that are as unobtrusive and as sensitive to different identities and forms of expression as possible.

In this paper, we report on the findings of a preliminary study analyzing COVID-19-related online health information provision for, and health information-seeking needs and behaviors of this population in order to generate insights that can be used to improve future health interventions and online information provision targeting this group. Our particular innovation is to use computational discourse analysis to examine online search results containing rich, wide-ranging digital media content such as quotes and anecdotes from older LGBTQ+ − identified individuals and practitioners, advice and recommendations from policymakers and healthcare experts, and research outcomes. This method allows for ongoing, immediate, and discursive data collection based on the results generated by queries being submitted through popular Internet search engines. In this way, it is building knowledge based upon questions being asked in real life about COVID and its implications for or effects on older LGBTQ+ community members, as well as the kinds of information and other discourse that is being accessed through those queries. Our findings to date support those of previous research: that older members of LGBTQ+ communities are experiencing important, even aggravated health and related social-economic disparities in comparison to the general population of older people. However, they also surface distinctive perspectives on resilience that are largely related to the support these individuals find inside the LGBTQ+ community that should be taken into account when healthcare and health information services are being designed. Although we recognize that there is a diversity of communities and experiences within the population of LGBTQ+ older adults, for this initial study, we chose to focus on the commonalities of LGBTQ+ older adults’ experiences, including disparities and resilience, rather than on the distinctive experiences of any specific subgroup. We argue, based on this limited study, that this provides proof-of-concept that computational discourse analysis could be a beneficial methodological tool, especially if used in conjunction with more traditional research methods such as surveys and interviews, in arriving at better understandings of the particular health and health information needs, behaviours and experiences of older LGBTQ+ community members. Future studies will refine this approach to provide more specific and fine-grained analyses of the experiences and needs of different groups within this population.

## Background

In 2011, the Institute of Medicine stated that “Lesbian, gay, bisexual, and transgender individuals have unique health experiences and needs, but as a nation, we do not know exactly what these experiences and needs are” [10]. More recent studies have recognized the need to ensure that the education of health professionals better supports working with LGBTQ individuals, as well as the need to understand better the intersections between ethnicity, age, and class [11–14]. Understanding and addressing these knowledge gaps and intersections have become even more critical under pandemic conditions. Older members of LGBTQ+ communities comprise a particular intersectional population that is prone to the health vulnerabilities, depression, stigmatization, and isolation associated with old age that make them at higher risk than the general population for potential effects of COVID [15]. At the same time they are also subject to health vulnerabilities that are the result of historical oppression, repression, and fear as LGBTQ+ community members [16, 17].

There are currently over three million LGBTQ+ Americans aged 50 years or older. 1.1 million aged 65 or older [18] (hereafter referred to as “elders”) suffer from various health and social disparities including pre-existing mental or physical health conditions, isolation, and systematic discrimination and stigmatization. The advocacy group Human Rights Campaign has called for greater awareness of how health vulnerabilities and economic disparities have compounded the impacts on LGBTQ+ communities of the global pandemic [19]. The Movement Advancement Project highlights yet another intersectional concern when it argues that there are special disparities for LGBTQ+ people of color as well as those with low income and that there is a “need for targeted assistance and explicit protections from discrimination” [20]. The effects of isolation as a result of social distancing can be more pronounced for LGBTQ+ elders, who are less likely to have children and more likely to live alone than their cisgender and heterosexual counterparts [21]. Dawson et al. (2021) argue that they may also be disproportionately affected by poverty since many LGBTQ+ elders work in the entertainment or service industries, which were among the first to be shut down during the initial wave of the pandemic. Moreover, LGBTQ+ elders may be less open to disclosing their sexuality or seeking help due to fear of judgment and stigmatization, making them more vulnerable to neglect and mistreatment in aging care facilities [22].

In addition to these health and economic disparities, LGBTQ+ elders may be disadvantaged and have specific concerns and behaviors in terms of both internet presence and use. During the pandemic, internet resources, social media platforms, and mobile apps have played prominent roles in something of a push-pull dynamic. They have been heavily used by governments and health and health information providers for the provision of health services and the dissemination of relevant information. The public has also turned heavily to social media and news services, both for information, debates, and news relating to COVID-19 and for social support to cope with the isolation and fears that have accompanied the pandemic and lockdown regimes. Overreliance on online sources may have a negative impact on the users, however. For example, multiple previous studies have indicated that social media use can have a mental health toll [23, 24] and, since the outbreak of the pandemic, that it has led to eroded public trust in its management and treatment of the virus [25]. As with the older general population, older LGBTQ+ community members may be less likely to be sufficiently technologically competent or equipped with the necessary devices to navigate social media platforms, apps, and other online resources. More specific to members of LGBTQ+ communities, many may be reluctant to share opinions and personal stories with strangers on the internet [26]. Conversely, however, precisely because of the above-outlined conditions, they may rely particularly heavily on online communication and information to search for information, send and receive emails, browse the news and take audio and video calls [27]. Magee et al. found similar ambivalent behaviors in their study of the information-seeking practices of LGBTQ+ youth, who displayed considerable interest in seeking sexual health information online but were concerned about stigma if they might be observed searching for information relating to LGBTQ+ or HIV topics [28]. Their results revealed not only significant interest in online sexual health information but also concerns about the perceived limitations of this promising method of sexual health promotion [28].

Although historically marginalized and stigmatized, the older LGBTQ+ population has demonstrated remarkable resilience. Multiple studies evaluated how older adults in LGBTQ+ communities applied innovative approaches to combat heterosexism and homophobia throughout the course of their lives [29, 30]. By resisting the obstacles they faced and trying to build new pathways, they are better positioned to cope with the challenges of aging [31]. In a study of 2560 LGBT adults ages 50 to 95, [32] Fredriksen-Goldsen also found that most reported being satisfied with their lives and were aging successfully with strong personal and social ties. These LGBTQ+ older adults’ social engagement and connectedness served as important strengths and were linked to positive health outcomes.

Library and information services are frontline providers of health information to health professionals and the general public and, we propose, would be key beneficiaries of the kinds of enhanced knowledge and insights that computational discourse analysis can provide. When designing and enhancing their information services and systems, it is essential that they understand as much as possible about the information needs, information-seeking behaviors, and barriers experienced by the populations they are targeting as well as how they come to trust not only information but also those who provide it. Hawkins et al. argue, however, that “health sciences librarianship has been slower than other areas of the profession in creating an evidence base covering the needs of its LGBTQ patrons” [33] even though an overarching critical librarianship movement inside health sciences libraries is” focused on interrogating and disrupting inequitable systems” [34]. Interestingly, the findings of a 2016 study of the information-seeking practices of LGBTQ+ health sciences information professionals themselves, about which little was previously known, were similar to those of the wider LGBTQ+ population in that they tended to reach out to other LGBTQ+ professionals “as more likely to have specialist knowledge, or through concern that non-LGBTQ librarians may be more likely to react in a stigmatizing or discriminatory way”. The same study also found that the librarians were less inclined to use a “chat” function for accessing online information, even though it was anonymous, and suggested the need for other kinds of information services that are better tailored to the concerns and behaviors of LGBTQ+ communities [35].

## Methods

The unique experiences of LGBTQ+ elders during COVID-19 have primarily been studied to date through interviews and surveys, which can have important logistical limitations in the scope and number or type of participants [36]. With the aim of expanding the scope and methods of such research as well as exploring online information-seeking behavior and queries of LGBTQ+ elders, our study takes a different approach and applies computational discourse analysis to the results of online searches for health information for this population.

**Fig. 1:**
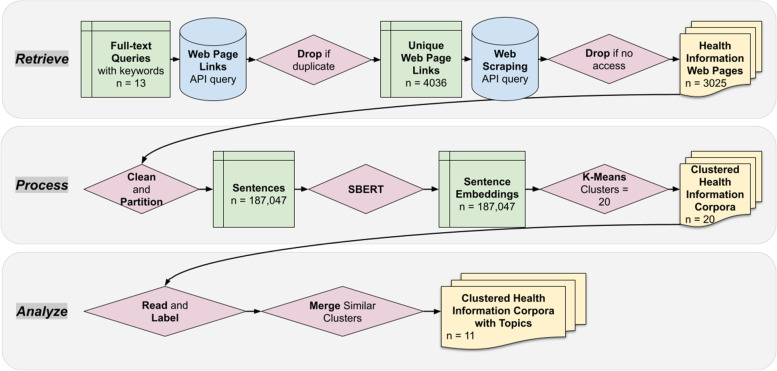
The workflow of computational discourse analysis. *Note:* The colour of each frame represents the category of the sub-step – green frames are in-step text sources, yellow frames are end-of-step text results, red frames are actions of computational decision-making or human analyses, and blue frames are API querying actions

Computational discourse analysis uses natural language processing (NLP) methods to automatically detect cohesion and local coherence that can help in making summative inferences about documents [37]. This is a method derived from data science that is increasingly being applied to harvest data and build archives from social media and World Wide Web pages [38, 39] and also to do predictive modeling [40]. As shown in Fig. 1, the study follows a workflow whereby online search data that is available through full-text searches on three widely used search engines – Google, Yahoo, and Bing – is retrieved, processed, and analyzed.

In this section, we introduce the materials referenced or contained in the online search results, the NLP methods used in the computational discourse analysis workflow, and our resulting disparities and resilience analytical framework.

### Description of materials

The Biden Presidential administration’s COVID-19 Health Equity Task Force recommends that federal, state, local, tribal, and territorial health departments collect data on sexual orientation and gender identity (SOGI) [41]. This recommendation responds to a scarcity of information or survey data that might be useful in studying health needs, care, and outcomes for LGBTQ+ elders. Many elders who were subjected to decades of stigmatization and discrimination remain reluctant to disclose their sexual orientation. For example, of the over 10,000 participants who responded to the 2020 Health and Retirement Survey (HRS), merely 3% answered the sexual orientation question [36]. Because of the lack of self-disclosure and large-scale survey data, it has been difficult to conduct health-related research on LGBTQ+ elders during the COVID-19 pandemic, a situation that in turn can lead to slower or less informed policy development and a lack of public awareness. Thus it is important to try to identify additional ways to build this data and expand its coverage. For our study, we have sought out data about LGBTQ+ elders and their COVID-19 health-related information needs and experiences based on publicly available online search results that can be digitally harvested and then analyzed. The information we have gleaned not only addresses some of the limitations of existing information and data resources but also encompasses a range of perspectives that can be made available for further study by interested parties ranging from policymakers and health providers and information professionals to LGBTQ+ elders themselves.

To obtain online search results relating to LGBTQ+ elders and information related to COVID-19, we used a straightforward keyword-based protocol. We first compile a list of 13 full-text queries using combinations of relevant keywords and abbreviations relating to older people, LGBTQ+, and COVID-19. These keywords could obviously be fine-tuned to develop queries for specific communities within the LGBTQ+ population, or indeed other populations altogether. In the data exploratory analysis stage, we experiment with “bisexual”, “bi”, “transgender” and “trans” as full-text queries but they resulted in no, very few, or duplicated records. To reduce API processing time, we chose the set of 13 queries shown in Table 1 because they were likely to retrieve the majority of online information related to LGBT+ and COVID-19.

**Table 1:** Keywords and Full-text queries for SERPAPI search

Keywords relating to age	Keywords relating to LGBTQ+	Keywords relating to COVID-19
**Keywords**		
elder	lgbt	covid
older	queer	coronavirus
senior	gay	
boomer	lesbian	
**Full-text queries**		
lgbt older covid	queer older covid	gay senior coronavirus
lgbt elder coronavirus	queer elder covid	gay older covid
lgbt older coronavirus	queer older coronavirus	lesbian elder covid
lgbt senior covid	queer elder coronavirus	
lgbt senior coronavirus	lgbt boomer covid	

Table 1 shows the stemmed keywords (i.e., at their minimum length to be unambiguously meaningful and retrieve the most possible results when used in full-text queries). To select the most frequently used queries, we empirically evaluate the combinations of keywords in a sample of results of online searches conducted on Google. As Table 1 shows, we find 13 frequently used combinations.

We then use the web scraping API, SerpApi,^1^ to access the top 50 pages of search result links for queries made through Google, Yahoo and Bing on one specific day, June 4, 2022, using those keywords. We remove duplicate result links and then retrieve the contents of the result links using the Request^2^ and BeautifulSoup^3^ packages in Python to create a corpus of online search data related to LGBTQ+ elders and COVID-19. We further clean this corpus by removing any special characters that might be irrelevant to or cause errors in the analysis process. The resulting final data corpus contains 187,047 sentences drawn from 3025 documents, including but not limited to news articles, blog posts, social media posts, and government documents. Table 2 shows examples from the data archive, where the schema includes title, source, date, article snippet (abstract), and article full text (not shown in the table).^4^

**Table 2:** Example online health information of LGBTQ+ elders and COVID-19

Title	Source	Date	Article Snippet
COVID-19 is hitting older LGBTQ adults especially hard	https://healthjournalism.org/blog/2020/04/covid-19-is-hitting-older-lgbtq-adults-especially-hard/	04/22/2020	He said 59% of LGBT older people report that they lack companionship, and 53% feel isolated. The situation for rural elders is even more tenuous. In a 2018 study, AARP found access to LGBT resources and LGBT senior resources was significantly reduced in rural areas. This crisis puts all of these issues in sharper focus, said Wilkinson.
Lesbian, Gay and Bisexual Adults and COVID-19 Risk	https://www.aarp.org/health/conditions-treatments/info-2021/lgb-community-increased-covid-risk.html	02/28/2021	A recent report commissioned by AARP New York and SAGE similarly found that older LGBTQ New Yorkers face steeper barriers to health care than their non-LGBTQ counterparts that put them at risk of experiencing COVID-19 more acutely, including: poor physical and mental health, alcohol and tobacco use, HIV, and cancer.
Why this 90-year-old man decided to come out as gay during the pandemic	https://www.washingtonpost.com/lifestyle/2020/06/30/load-has-been-lifted-why-age-90-this-man-decided-come-out-gay/	06/30/2020	Kenneth Felts spent the last 90 years of his life not telling anyone he was gay, but during the coronavirus pandemic everything changed.
US gay, lesbian adults have higher COVID vaccination rates	https://www.nydailynews.com/coronavirus/ny-covid-gay-lesbian-vaccination-rates-higher-than-heterosexual-adults-cdc-20220203-f2nxcezjt5bvrjuq4jif5xdqqa-story.html	02/03/2022	According to a report released by the Centers for Disease Control and Prevention, lesbians and gay men aged 18 and older reported higher vaccination rates.
Impact of the COVID-19 pandemic on the LGBT community	https://en.wikipedia.org/wiki/Impact_of_the_COVID-19_pandemic_on_the_LGBT_community	06/01/2022	More than 220 gay pride celebrations around the world were canceled or postponed in 2020, and in response a Global Pride event was hosted online.

### NLP methods

Due to the complexity and the sheer size of this corpus, it would be difficult for a human to read all 3025 documents and 187,047 sentences and accurately derive knowledge from them. NLP methods, however, can process with both speed and granularity and have demonstrated potential in research relating to online information and health information. By using NLP to effectively analyze complex semantic meanings and hidden emotions contained in large-scale online information corpora, researchers have been able to identify insights for such purposes as online marketing, customer relationship management, and monitoring public opinions [42]. For example, Liu and Toubia used semantic methods to estimate consumer content preferences from online search results [43], and Yin et al. employed Machine Learning approaches to identify controversial speech related to COVID-19 on Twitter [44]. NLP has also been gaining popularity in the fields of healthcare, especially with the increasing accessibility of Electronic Health Records data. NLP algorithms are used to facilitate the access and retrieval of valuable healthcare information [45] and detect patterns for disease identification [46]. Additionally, researchers have been leveraging NLP methods and online search information to study health and healthcare-seeking behaviors. Studies have proposed using Artificial Intelligence and NLP methods to identify, track and archive health behaviors from information streams on the Internet and social media sources [47, 48]. By leveraging such rich online health information behavior and resources by means of powerful computational techniques, therefore, researchers can better understand that behavior and associated knowledge consumption, as well as generate recommendations about concerns such as surveillance, health communication, and knowledge translation in online contexts.

In this study, we use NLP and related semantic measurements to make summative inferences about online health information and related context. Because of the variety of topics in each item retrieved through our online search, we conduct sentence-level processing, which enables topical clustering of health information relating to LGBTQ+ elders.

After cleaning and preprocessing, we first use Sentence-BERT (SBERT), a transformer-based pre-trained NLP model that applies a deep understanding of how language works, to derive semantically meaningful sentence embeddings that can be efficiently compared using similarity measuring methods [49]. SBERT is based on a series of previous methods used in deep learning and NLP. Transformers use attention mechanisms to connect the encoder and decoder in modeling sequence data, for example, text data, to achieve better quality and less processing time [50]. BERT is an NLP model based on the idea of transformers that pre-trains deep bidirectional representations from the unlabeled text [51]. SBERT modifies the pre-trained BERT network and significantly reduces computing time for the task of sentence similarity measurement by integrating the Siamese network. SBERT can derive semantically meaningful sentence embeddings that can be used for clustering and topic modeling. Currently, SBERT has limited but emerging usage in healthcare, particularly with applications in electronic health records (EHR) data [52]. Our implementation takes advantage of the open-domain attribute of SBERT to enable broader discovery and more inclusive clustering of not only health information but also the related social-economic context. In particular, we use the Python implementation^5^ of SBERT based on the pre-trained model ‘all-MiniLM-L6-v2’,^6^ which maps sentences in our corpus to a 384-dimensional dense vector space that is suitable for tasks including clustering and semantic searching.

We then use K-Means clustering to bring together sentences with similar semantic topics, implementing it using the scikit-learn package in Python.^7^ We use Lloyd’s K-Means clustering algorithm because of its simplicity and low time complexity in processing hundreds of thousands of sentence embeddings with high dimensions [53]. As Algorithm 1 shows, it first randomly initiates a number (a predefined hyperparameter) of centroids in the 384-dimension vector space and randomly assigns a centroid label to each of the 187,047 sentences embedding vectors. It then uses Euclidean distance as a measurement to recursively update the centroid assignments. The updating loop stops when the centroid assignment to each of the sentences no longer changes and we keep the assigned centroid as their cluster labels. Regarding the number of centroid hyperparameters that we need to predefine, we experiment with three different numbers of clusters: 10, 20, and 50. Because 50 clusters result in sparse clusters and 10 clusters lead to overly large clusters containing several topics in each of them, we decide upon 20. While the sentence embeddings remain the same, the clustering results can be slightly different with different statuses (seeds) of randomness. Since the order and the trivial difference among the clusters of sentences do not matter for our research on discourse, we pick one of the results for demonstration in the Results section.

**Figure:**
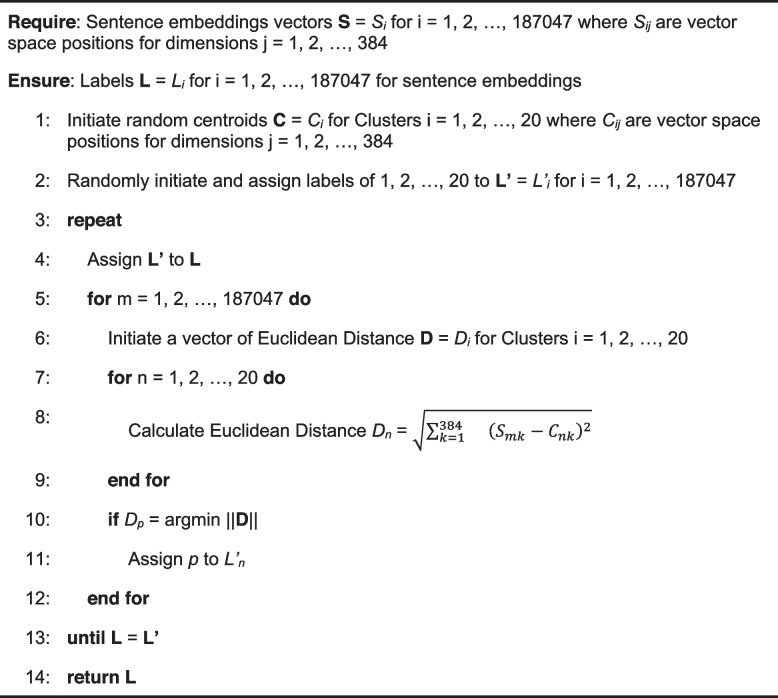
**Algorithm 1.** Lloyd’s K-Means algorithm for clustering sentence

### The disparities and resilience (D&R) analytical framework

The current analytical frameworks applied to underrepresented or minority communities are usually developed from policymakers’ perspectives. For example, the Vulnerability to Resilience (V2R) framework specifies key areas for analysis and action to address the diverse factors that contribute to vulnerability in communities during disasters [54]. Similarly, research regarding LGBTQ+ elders often specifically focuses on the activities of government institutions. For instance, the analysis of resilience and disparities among LGBT older adults addresses the commitments and policy research of U.S. national institutions, including the National Institutes of Health, the Institute of Medicine, and the Centers for Disease Control and Prevention [29].

To analyze the materials collected from an extended internal perspective that highlights LGBTQ+ elders and their community, we expand the analytical framework to include the analysis of information provision, behaviors, and needs of LGBTQ+ elders. Specifically, we implement a Disparities and Resilience (D&R) framework that we have devised.

**Fig. 2:**
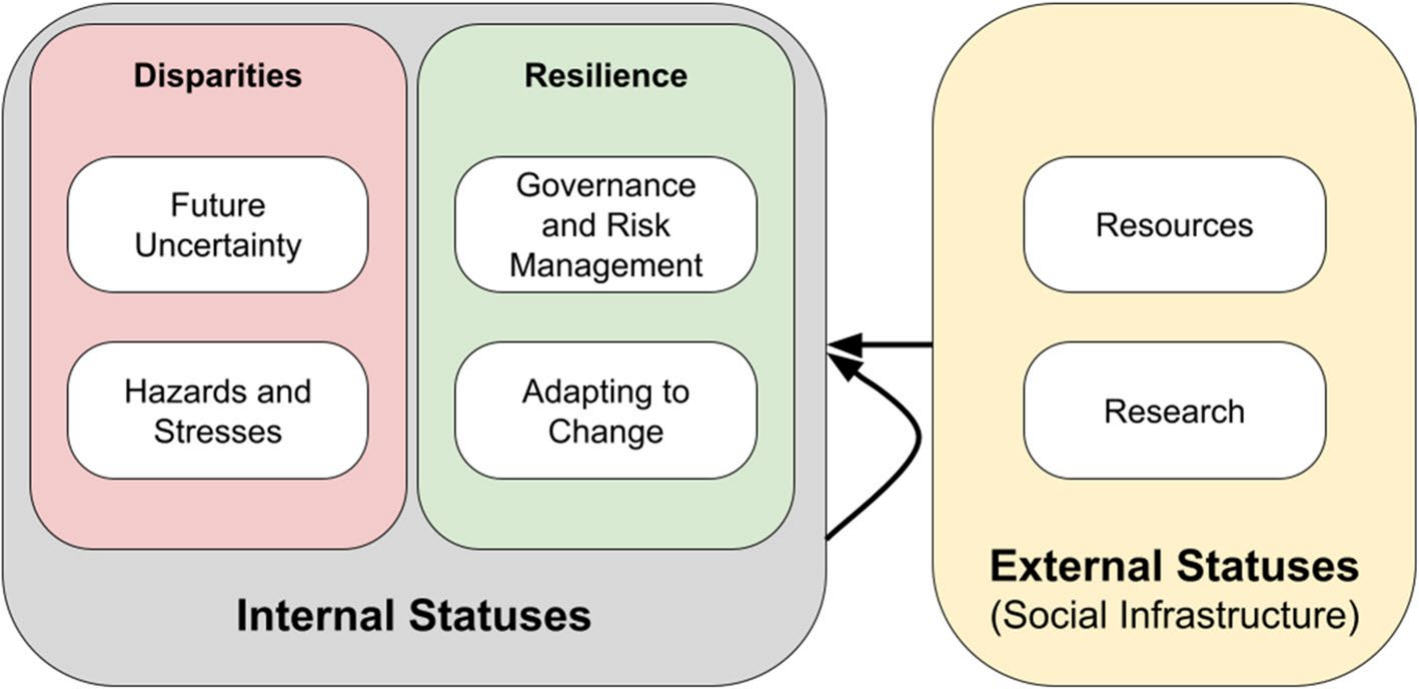
The disparities and resilience (D&R) framework. *Notes:* 1) The internal statuses are aspects of LGBTQ+ elders themselves. The external statuses relate to social infrastructure. 2) The arrows represent the analytical relationship between source and application. Here, we use both external and internal statuses to analyze Disparities and Resilience in the internal statuses of LGBTQ+ Elders

As Fig. 2 shows, the D&R framework first demonstrates how the mixed outcomes of disparities and resilience are co-existing characteristics among the LGBTQ+ elders population: “Disparities” here include immediate “Hazards and Stresses” and “Future Uncertainty”, while “Resilience” includes “Governance and Risk Management” and “Adapting to Change”. We argue that LGBTQ elders are not only passively influenced by policy changes, services, and intervention developments (i.e., by social infrastructure), but also actively participate in and organize their own communities with unique networks of support intended to improve their current and future well-being (i.e., internal statuses). The analysis based on the D&R framework accounts for both external and internal information and therefore provides more nuanced perspectives that we hope will be useful to decision-makers and information service providers in both governmental and non-governmental (including community) organizations.

## Results

For the analytical results, as the “Analyze” step in Fig. 1 indicates, two annotators read and analyze the 20 clusters of the health information corpus generated by the NLP method. We then merge eight of them into clusters with similar topics, resulting in 12 clusters with coherent topics that both annotators agree on. We conduct intercoder reliability checks through the calculation of percent agreement and obtain a high agreement rate (85%) that confirms the internal consistency and validity of this study.

**Fig. 3:**
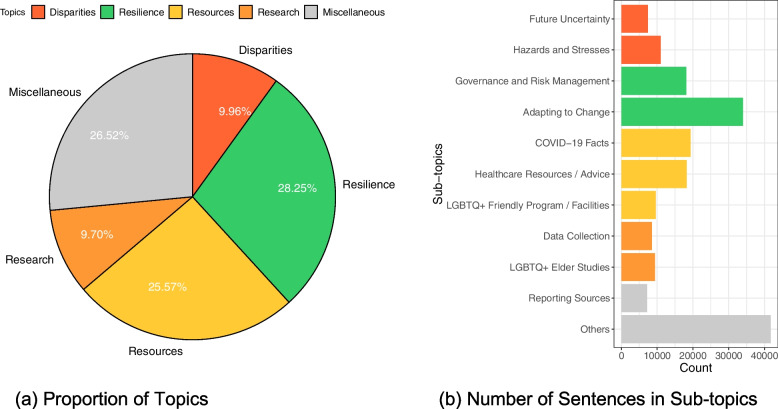
Overview of Topics and Sub-topics in the Health Information Corpora. **a** Proportion of Topics. **b** Number of Sentences in Sub-topics

Our analysis results in five topics – Disparities, Resilience, Resources, Research, and Miscellaneous – that correspond to the internal and external statuses of LGBTQ+ elders in the D&R framework. We further label each cluster with a sub-topic, resulting in 11 sub-topics (Table 3). As Fig. 3 shows, the two largest clusters in the online corpus relating to LGBTQ+ elders are both above a quarter of the corpus, including the topics of Resilience, which contains 52,138 sentences making up 28.25% of the corpus, and Resources, which contains 47,187 sentences making up 25.57% of the corpus, while the topics of Disparities (18,377 sentences) and Research (17,905 sentences) are both about one-tenth of the corpus. By definition, a Miscellaneous topic defies close definition and easy analysis. Table 3 and Fig. 3 provide an overview of about a quarter of the corpus and suggest the diversity and complexity of information retrieved through Internet searches.

**Table 3:** Clustered health information corpora with topics

Topic	Sub-topic	Cluster(s)
**Disparities**	Future Uncertainty	4
	Hazards and Stresses	11
**Resilience**	Governance and Risk Management	8, 10
	Adapting to Change	1, 9, 12
**Resources**	COVID-19 Facts	5, 15
	Healthcare Resources / Advice	3, 18
	LGBTQ+ Friendly Program / Facilities	14
**Research**	Data Collection	2
	LGBTQ+ Elder Studies	16
**Miscellaneous**	Reporting Sources	6
	Others	7, 13, 17, 19, 20

### Disparities

As already noted, the discourse on the topic of “Disparities” has two main themes – “Future Uncertainty” and “Hazards and Stresses”. Table 4 shows examples of sentences captured within the Disparities topic. The “Future Uncertainty” sub-topic mainly contains subjective expressions of frustration, with a focus on worries about future feelings or the long-term impacts of COVID-19. The “Hazards and Stresses” sub-topic contains references to a variety of negative impacts of COVID-19, ranging from general effects on sub-groups in the LGBTQ+ communities to specific aspects that affect daily life such as job security and mental health.

**Table 4:** Example sentences of disparities with topics and clusters

Topic	Sub-topic	Examples	Cluster
**Disparities**	**Future Uncertainty** (expressing frustration)	I wasn’t sure—about the future, about what to do now.	4
		The possibility that I couldn’t be with him in the hospital was very difficult.	4
		“They’re there, but they’re just hidden.	4
		“We don’t want to be left behind.	4
		“They often don’t have a community that will take care of them.”	4
		“I felt belittled, and I was thinking: what would happen to us if we were living on our own and renting a place and are excluded?”	4
	**Hazards and Stresses** (including attitudes towards Governance and Livelihoods)	In addition to the physical health impacts of COVID-19 on LGBT elders, mental health is a key concern.	11
		They have also suggested that older lesbians face particular ongoing challenges in relation to anti-lesbian discrimination at the personal, social, and wider levels.	11
		When asked about the queer media niche in particular, and the affects coronavirus will have short and long term on these individuals and businesses specifically, she says, “people in queer media are really scared.”	11
		Experiencing discrimination and stigma throughout one’s life leads to high levels of mistrust in the medical profession and government among LGBT and other diverse elders.	11
		COVID-19 has resulted in job losses and financial insecurities, especially for marginalized populations.	11

### Resilience

The discourse on the topic of “Resilience” also has two main themes – “Governance

and Risk Management” and “Adapting to Change”. Table 5 shows examples of sentences contained within the Resilience topic. The “Governance and Risk Management” sub-topic positively underlines the value of community engagement in public health activities for and by LGBTQ+ elders. The “Adapting to Change” sub-topic further demonstrates, through personal recollection or quotes, specific mental and daily routine changes and adaptations.

**Table 5:** Example sentences of resilience with topics and clusters

Topic	Sub-topic	Examples	Cluster
**Resilience**	**Governance and Risk Management** (with Diversity and for Security)	Some even reported an improved quality of life, better personal relationships and increased neighborly support.	8
		“Community plus public health is magic.”	8
		Vaccine: While LGBT people report wanting to get vaccinated at a similar pace as non-LGBT people, a greater share of LGBT adults see doing so as part of everyone’s responsibility to protect the health of others (75% v. 48%), while greater shares of non-LGBT people see vaccination as a personal choice (49% v 24%).	10
	**Adapting to Change** (through personal recollection or quotes)	“I support the protests 1000%, but I think they are probably pretty good breeding ground for the virus,” he said.	1
		And she was 80 this year — we had a birthday party on Zoom — all of her lesbian and non-lesbian friends joined in to celebrate.	1
		He continues to receive his weekly food delivery from Meals on Wheels and relies on a friend to help him with his shopping needs.”We have to deal with it on a day-to-day basis and do what we can to protect ourselves,” said Longen.	1

### Resources and research

The topics of “Resources” and “Research” cover the status of external support to the LBGTQ+ elders. Table 6 shows examples of sentences contained within both topics: The topic “Resources” provides COVID-19-related facts, healthcare advice, and information on inclusive public health programs; “Research” includes data collection and analytical results regarding health information for LGBTQ+ elders during COVID-19. These two topics provide information on social welfare and external challenges for the internal statuses of the LBGTQ+ elder population.

**Table 6:** Example sentences of resources and research with topics and clusters

Topic	Sub-topic	Examples	Cluster
**Resources**	**COVID-19 Facts**	Persons aged 12 years and older should receive 2 doses at least 3–8 weeks apart.	5
		Roughly half (51%) reported following the news very closely in mid-March, a figure that grew to 57% later that month.	5
		Over 1.1 million are 65 and older, according to SAGE.	5
	**Healthcare Resources / Advice**	Coronavirus: latest news and government advice	3
		A drive-thru coronavirus testing center in Denver.	3
	**LGBTQ+ Friendly Program / Facilities**	The program is a multi-part online and phone text-based suite of services designed to reduce the spread of COVID-19 and its negative consequences among Wisconsin’s transgender and non-binary community members and their partners, loved ones, friends, allies and contacts.	14
		SAGE has an LGBT elder hotline, 877–360-LGBT, for older adults to have confidential discussions with caring people to get information on supportive agencies and basic needs.	14
		LGBTQ charities have pivoted to online, telephone and video platforms to ensure people in need of psychological support continue to receive it.	14
**Research**	**Data Collection**	Finally, smaller sample sizes of LGBT persons might have yielded low statistical power to detect differences by sexual orientation and gender identity in stratified analyses.	2
		We’re continuing to learn more about long Covid, but there’s still a lot we don’t know about the condition and how to help people who are suffering.”	2
		Unlike the virus that spawned it, long COVID is a chronic illness with a range of symptoms and there is no clear test for it.	2
	**LGBTQ+ Elder Studies**	Opening Doors collaborates in a new study that investigates social care assessments of older LGBTQ+ people in England	16
		By combining a series of three questions, the survey allows researchers to compare the recent experiences of the LGBT population to other adults.	16

## Discussion

To study health care needs and health outcomes of LGBTQ+ elders, researchers have tended to rely on survey data and interviews. However, data completeness and interviewee recruitment are difficult to guarantee under pandemic conditions. Our research uses a method that does not require in-person interaction with subjects and, although for the sake of this study we only retrieved data from 1 day, has the potential to gather very large, timely, and time-based samples of personally contributed queries, reflections, and responses as well as information issued by official institutions. By analyzing this data from multiple perspectives – as seen and addressed from outside the LGBTQ+ elder population (the external social infrastructure) and internal to that population (their internal status) it is possible to surface the mixed and distinctive effects of the pandemic and pandemic measures on this population. From our small study, this panoramic view suggests both aggravated disparities and strengthened resilience of LGBTQ+ elders during COVID-19. More specifically, it indicates that the pandemic disproportionately impacts LGBTQ+ elders but that they have also adapted quickly, both mentally and technologically. While further studies need to look more closely at specific communities within this population, our study indicates that it is important for policymakers and service providers to be aware of both the disparities and resilience of LGBTQ+ elders during COVID-19 and develop health interventions and information services accordingly.

### Aggravated disparities: COVID-19 has disproportionately negative impacts on the health of LBGTQ+ elders

Clusters 11 and 14 in our online health information corpus suggest that the COVID-19 pandemic has compounded disparities affecting the immediate and long-term health of LGBTQ+ elders. Like all older people in the general population, LGBTQ+ elders deal with the immediate physical health risks and mental stress of, as well as uncertainty about future health conditions that might result from catching or recovering from COVID-19. However, when seeking COVID-19-related health information and support, there is insufficient help that is informed about and responsive to their particular concerns and situations.

As Cluster 11 indicates, the internal statuses of LGBTQ+ elders are influenced by and are worrying about the COVID-19 pandemic, a situation that can lead to both immediate physical health impacts and mental stresses (Table 4). Because of the historical and continuing discrimination against and stigmatization of LGBTQ+ communities, many LGBTQ+ elders are less willing to express their needs for health care and if they do not have families, they may be particularly vulnerable to isolation during quarantines and lockdowns. Unequal job losses and financial insecurities due to COVID-19 also create further health disparities for LGBTQ+ elders, especially when public healthcare systems are more likely to be overwhelmed during the pandemic. As Cluster 4 shows, some LGBTQ+ elders “weren’t sure about the future” and “don’t want to be left behind”, reflecting general worries about their health status in the future. Others worry about being unable to accompany their partners to the hospital if they get COVID. Quotes from LGBTQ+ elders, healthcare practitioners, and caretakers demonstrate health disparities and often anticipate negative concerns about LGBTQ+ elders’ future health. One healthcare provider mentioned the lack of support network among LGBTQ+ elders: “they often don’t have a community that will take care of them” and an LGBTQ+ elder expressed fear of housing instability, saying that “I felt belittled, and I was thinking: what would happen to us if we were living on our own and renting a place and are excluded”. Coupled with a lack of social support systems and adequate healthcare accommodations [55, 56], these internal statuses of LGBTQ+ elders show aggravated disparities in their immediate and long-term well-being due to COVID-19.

Based on the data analysis, external resources during COVID-19 are seemingly abundantly available to LGBTQ+ elders. For example, Cluster 14 provides online health information on LGBTQ+ friendly programs, including information on programs developed by the Froedtert & MCW health network and SAGE (Table 6). These programs are carefully designed and provide not only online options but also traditional contact channels through phone or text for LGBTQ+ elders. However, such programs often operate on a smaller scale and nationwide policy support is lacking. In the US, 30 states do not have laws explicitly prohibiting housing discrimination based on sexual orientation and gender identity [57, 58]. LGBTQ+ and senior-living community-dwelling elders from these states might be more vulnerable to discrimination and abuse. Additionally, many federal elder service programs either fail to recognize LGBTQ+ elders as a “population of greatest social needs” or suffer from a limited budget and inconsistent funding [55, 59]. Thus, LGBTQ+ elders are less likely to receive competent and reliable services from federal or local organizations.

### Strengthened resilience: LBGTQ+ elders make quick mental and technological adaptations to COVID-19

In our online health information corpus, Clusters 1, 8, and 10 show the strengthening resilience of LGBTQ+ elders during COVID-19. At the same time, Clusters 2 and 16 indicate that current research is not fully aware of such resilience or the unique internal strength of the LGBTQ+ elders population.

From lifestyle adaptation to mindset change, the data suggest that LGBTQ+ elders have both relied on and strengthened their community-based support network during the pandemic (Table 5). Cluster 1 shows both first-person recollections and interview quotes of LGBTQ+ elders and how they adapted their activities. For example, a birthday meet-up of an LGBTQ+ elder was made virtual via real-time online video technology and large in-person gatherings were avoided. During quarantine, one LGBTQ+ elder, Logen, reported learning to use food delivery for daily supplies. Such community-reported health information-seeking and lifestyle adaptation behaviors also align with recent studies that indicate that LGBTQ+ elders in fact have a higher proportion of using digital technologies to connect with families and friends (75%) compared to heterosexual adults (55.9%) [60, 61]. Through time, such successful adaptation has transformed into spontaneous but systematic governance and risk management awareness, and their resilience further strengthens the LGBTQ+ elder population. For example, Cluster 10 refers to higher vaccination rates, and Cluster 8 demonstrates improvement in the quality of life and health expectations in the LGBTQ+ elderly population after COVID-19. These discourses confirm that although LGBTQ+ adults face more barriers to vaccination access such as race, income, and other social determinants, they show a higher proportion of vaccination rate compared to their heterosexual counterparts (85% VS 76%) [62].

While the findings of our discourse analysis support those of a few recent studies, as an important part of the social infrastructure, in general, current research is not fully aware of the strengthened resilience of LGBTQ+ elders because of the earlier discussed difficulties in data collection and research methods (Table 6). This lack may impede fast and effective policy development and external interventions. As Cluster 2 indicates, the sample sizes in recent surveys related to COVID-19 and LGBTQ+ elders are insufficient for thorough analysis. In terms of chronic data on COVID-19-related health problems, for example, data on long COVID is also unavailable because of the lack of testing techniques by which long COVID can be identified. Thus, it is currently difficult for researchers to make a robust analysis of the health outcomes for LGBTQ+ elders who have had COVID-19. As Cluster 16 shows, many research projects instead focus on using “recent experiences” gathered through surveys or interviews for analysis, which are comparatively less extensive or current than the data contained in the online health information corpus that we have captured.

### Limitations

There are some data and methodological limitations in our study. First, some data are missing from the corpus we have created, although the proportion is low (less than 25%). This is due to website access restrictions that mean that some search links in pages, although they can be accessed manually, cannot be scraped using automatic tools through web APIs.

Second, our analysis of online search contents combines computational and human processes, which involves both the effects of computing randomness and of human judgments. To validate that the scraping process has behaved as expected, we have employed a manual process of content relevance checking, which is laborious. To ensure that the 3025 items in the corpus were relevant to LGBTQ+ elders, we sampled 100 of them and inspected their content. We used two human coders to ensure inter-coder reliability. Of the 100 samples, more than 80% were related to two of the topics in LGBTQ, elders, and COVID. Our manual check suggests that the majority of the scraped search results are either completely or highly related to the focus of the study, while some may be of less direct relevance. For simplicity, we have still included them even though our analysis is not based on them. Again we are currently using manual review to analyze the topics of clustered sentences and check for internal reliability and validity. We are confident that the majority of the sentences have topical coherence, although some of the sentences can be irrelevant. We thus have to regenerate clusters and focus on the sentences that are consistently grouped together. This process may include some unavoidable randomness in topic clustering. Lastly, as already alluded to, the analytical unit for our computational discourse analysis may lead to potential limitations regarding analytical granularity. Because of the de-contextualized nature of the sentences we used in the study, it is difficult to differentiate the subjects of the sentences by specific subgroups or communities within the LGBTQ+ elder population (e.g., transwoman, bisexual male, etc.,) or by demographic characteristics (e.g., race, socio-economic status). As a result, our method tends to homogenize the experiences of different communities of the LGBTQ+ population, thus policy implications focus on the overlapping needs rather than differences between LGBTQ+ elder communities. At the same time, while using a sentence as the analytical unit can result in the loss of some contextual details, we believe that sentence-level granularity better distinguishes the different layers of information in an article. Thus, we preserve the interpretable sentence structure that is best suited for the main analytical objective: identifying common disparities and collective resilience of the LGBTQ+ elder population.

## Conclusions and future work

The COVID-19 pandemic is both a global health and an information crisis that is simultaneously an infodemic [63] that has had a disparate impact on different populations. LGBTQ+ elders have been especially negatively affected due to a lack of awareness and knowledge about their information and healthcare needs, their information-seeking behaviours, and their current support structures and networks.

Using computational discourse analysis of the results of Internet queries made using three popular search engines, we observe that the pandemic appears to aggravate health disparities in LGBTQ+ elders, but that this diverse and large community also shows strengthened resilience as well as strong use of digital information resources. On the one hand, the external status of resources and research on the population indicates limited public awareness and targeted support for LGBTQ+ elders during COVID-19. On the other hand, many LGBTQ+ elders have successfully adjusted to the “new normal” because of their distinctive internal statuses, such as their experience with the AIDS pandemic, the affordances of digital media in terms of privately seeking relevant information, and strong interpersonal connections within their community.

Our study provides evidence that methodological innovation in gathering and analyzing digital data relating to overlooked, disparately affected, and socially and economically marginalized intersectional communities such as LGBTQ+ elders can result in better external and self-knowledge of these populations. Specifically, it demonstrates the potential of computational discourse analysis to surface hidden and emerging issues and trends relating to a community that has important concerns about public exposure, and to triangulate data gathered through more traditional mechanisms such as surveys and interviews in highly timely and automated ways.

For the next phase of our research, we plan to utilize topic modeling methods, such as BERTopic [64], to generate topic clusters and automatically extract frequently occurring words to represent the topic of each cluster. Additionally, we plan to combine the current text analysis method with existing data gathered through interviews and survey results, such as the 2020 Health and Retirement Survey (HRS) [36], to better understand and categorize LGBTQ+ elders’ resilience during the COVID-19 pandemic. We hope that this will further demonstrate the potential of using such mixed methods to uncover other aspects of strengthened resilience that were previously overlooked, and thereby inform future decisions in policy-making and healthcare and health information service provision, as well as promote additional community networking and other internal support mechanisms.

## Data Availability

The dataset analyzed during the current study is available at 10.3886/E176221V1.

## Abbreviations

BERT: Bidirectional encoder representations from transformers
BERTopic: A topic modeling technique that leverages BERT
COVID-19: Coronavirus disease, an infectious disease caused by the SARS-CoV-2 virus
D&R: Disparity and Resilience analytical framework
NLP: Natural language processing
SARS-CoV-2: Severe Acute Respiratory Syndrome Coronavirus-2
SBERT: Sentence-BERT, sentence embeddings using Siamese BERT networks
V2R: Vulnerability to Resilience analytical framework

## Footnotes

1 https://serpapi.com/

2 https://requests.readthedocs.io/en/latest/

3 https://www.crummy.com/software/BeautifulSoup/bs4/doc/

4 Our dataset of online health information of LGBTQ+ elders during COVID-19 is available at 10.3886/E176221V1

5 https://www.sbert.net/

6 https://huggingface.co/sentence-transformers/all-MiniLM-L6-v2

7 https://scikit-learn.org/stable/modules/generated/sklearn.cluster.KMeans.html
